# Occupational Exposure to *Pfiesteria* Species in Estuarine Waters Is Not a Risk Factor for Illness

**DOI:** 10.1289/ehp.8627

**Published:** 2006-04-18

**Authors:** J. Glenn Morris, Lynn M. Grattan, Leslie A. Wilson, Walter A. Meyer, Robert McCarter, Holly A. Bowers, J. Richard Hebel, Diane L. Matuszak, David W. Oldach

**Affiliations:** 1 Department of Epidemiology and Preventive Medicine and; 2 Department of Neurology, University of Maryland School of Medicine, Baltimore, Maryland, USA; 3 Institute for Human Virology, University of Maryland Biotechnology Institute, and Department of Medicine, University of Maryland School of Medicine, Baltimore, Maryland, USA; 4 Maryland Department of Health and Mental Hygiene, Baltimore, Maryland, USA

**Keywords:** commercial fishermen, dinoflagellates, environmental toxins, neuropsychological testing, occupational health

## Abstract

**Background:**

Exposure to the dinoflagellate *Pfiesteria* has, under certain circumstances, been associated with deficits in human learning and memory. However, uncertainties remain about the health risk of chronic, low-level exposures (as seen among occupationally exposed commercial fishermen), particularly in light of studies suggesting that *Pfiesteria* strains are widespread in the estuarine environment in the U.S. mid-Atlantic region.

**Methods:**

We selected an initial cohort of 152 persons, including 123 persons with regular, occupational exposure to the Chesapeake Bay; 107 of the cohort members were followed for the full four summer “seasons” of the study. Cohort members were questioned biweekly about symptoms, and data were collected about the areas of the bay in which they worked. These latter data were matched with data on the presence or absence of *Pfiesteria* in each area, based on polymerase chain reaction analysis of > 3,500 water samples. Cohort members underwent neuropsychological testing at the beginning and end of each summer season.

**Results:**

No correlation was found between work in an area where *Pfiesteria* was identified and specific symptomatology or changes on neuropsychological tests.

**Conclusions:**

Although high-level or outbreak-associated exposure to *Pfiesteria* species (or specific strains within a species) may have an effect on health, routine occupational exposure to estuarine environments in which these organisms are present does not appear to pose a significant health risk.

In the summer of 1997, a group of watermen (commercial fishermen) working on the Pocomoke River on the eastern shore of the Chesapeake Bay in Maryland developed a pattern of neuropsychological deficits marked by difficulties in learning and memory (Grattan et al. 1998). Initial deficits were profound, with affected individuals scoring 2–3 standard deviations below national norms on standardized tests, but resolved within 3–6 months of cessation of exposure to the river. Affected watermen had had constant, high-level occupational exposure to areas of the Pocomoke River where the dinoflagellate *Pfiesteria* (Burkholder et al. 1992) had been identified in association with several fish-kill events, and it was hypothesized that these river exposures contributed to the observed health effects (Grattan et al. 1998; Morris 2001). There were also suggestions that isolated, acute exposure to affected waterways in the midst of a fish-kill event could elicit a flu-like syndrome, albeit without accompanying changes in neurocognitive test performance (Haselow et al. 2001). Neuropsychological deficits similar to those seen among persons with constant, high-level exposure to the Pocomoke River had been previously observed after laboratory exposure to *Pfiesteria* (Glasgow et al. 1995; Schmechel and Koltai 2001) among persons working in the laboratory of J. Burkholder, who had initially described the organism (Burkholder et al. 1992). Studies by Levin and colleagues involving parenteral inoculation of rats with material from *Pfiesteria* cultures provided further support for the idea that exposure to *Pfiesteria* resulted in deficits in new learning and memory (Levin 2001; Levin et al. 2003).

With the subsequent demonstration that *Pfiesteria* is a common inhabitant of estuarine waters in the mid-Atlantic region and beyond (Jakobsen et al. 2002; Rublee et al. 2001), concerns arose about the possible health impact of chronic occupational exposure to *Pfiesteria* species. Although there are anecdotal reports that watermen working in areas where *Pfiesteria* were known to be present had non-specific health complaints, there has not been clear, objective documentation of the presence or absence of health effects associated with regular occupational exposure. In a small case–control study (22 cases, 21 controls) from North Carolina, no association was found between exposure and health effects, except possibly for a deficit in visual contrast sensitivity (Swinker et al. 2001); however, the study relied on fish health as a marker for the presence of *Pfiesteria*. Larger cohort studies funded by the Centers for Disease Control and Prevention (CDC) have been conducted in North Carolina and Virginia (Moe et al. 2001); the North Carolina study again used fish health as its marker for exposure, whereas the Virginia study used a combination of fish health and molecular data. We report here the results of a 4-year study (1999–2002) of a cohort of “high-risk” watermen and community controls in Maryland, in which symptoms and neuropsychological changes were linked with environmental exposure to *Pfiesteria* species as assessed by molecular methods.

## Materials and Methods

### Recruitment methods

Initial recruitment was based on a random selection of candidates from the 1997 Maryland Department of Natural Resources (DNR) Commercial Fisheries Licensure list, as stratified by age and ZIP code; recruitment was restricted to watermen living in counties/ZIP codes along the eastern shore of the Chesapeake Bay. Each participant had to average ≥10 hr/week on Maryland Chesapeake waters or tributaries. Each had to be healthy, with no self-reported history of past head injury, stroke, dementia, or drug or alcohol abuse. When difficulties were encountered in reaching the desired number of participants, this approach was modified to a “semi-open” recruitment process, to include referrals by previously enrolled participants. Initial efforts were also made to recruit community control participants (who had minimal contact with estuarine waters) using drivers’ license records to match to enrolled watermen by ZIP code, age, and education. All applicable human volunteer requirements were followed; the study was approved by the institutional review board (IRB) at the University of Maryland, Baltimore and the Maryland Department of Health and Mental Hygiene. All participants gave written informed consent before the study.

We enrolled 123 watermen and 29 controls, for a total of 152 participants. The average age was 47 years (range, 19–74 years); all participants but one were male. Forty-five (30%; 35 watermen, 10 controls) of the 152 were lost to attrition during the 4-year time period of the study. Of the 45 who dropped out of the study, 26 (58%) cited as their primary reason the inconvenience of the testing and paperwork required by the study; 4 (9%) moved out of the area, and 2 died. One hundred seven watermen participants who enrolled in the study in year 1 completed the full 4 years of follow-up. Among watermen, those who dropped out were slightly younger at the time of enrollment than those who stayed in the study (42.2 years vs. 48.8 years; *p* = 0.02, chi-square); otherwise, there were no significant differences between those who dropped out and those retained in the study. The two deaths were in the exposed group; in both instances, deaths were from causes that were independent of the exposures being evaluated in this study.

### Study design

**Figure 1:**
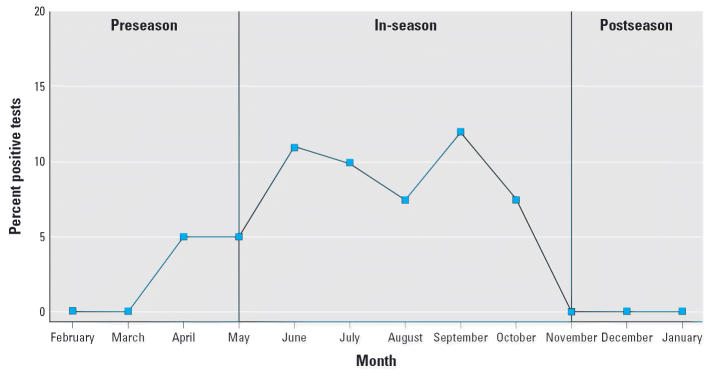
Percentage of all environmental tests showing a positive result for the presence of *Pfiesteria*, by month, for 1999–2002.

Data collection centered around the four summer “seasons” in 1999, 2000, 2001, and 2002. This reflects the patterns of occupational exposure of the watermen (whose on-the-water work year is generally restricted by weather to spring, summer, and fall) and is in keeping with our environmental surveys, which have shown that *Pfiesteria* demonstrate a clear seasonality, with organisms detected with increasing frequency in the water column during the summer and early fall, and then “disappearing” as winter begins (Figure 1). For the purposes of this study, each calendar year was broken into pre-season (February through April), active season (May through October), and postseason (November through January).

Biweekly monitoring was accomplished by self-reported logs or diaries. Every 2 weeks throughout the year, each participant was sent a log covering the previous 2-week period. The participant was asked to answer questions dealing with how many days he/she worked, where the work occurred, what type of fishing was engaged in, whether any type of fish “event” was witnessed, any symptoms experienced (see Appendix), and whether he/she was exposed to any type of known chemical toxicants. Symptom lists were based on symptoms reported in the 1997 Pocomoke River outbreak (Grattan et al. 1998), persons with exposure in the Burkholder laboratory (Glasgow et al. 1995; Schmechel and Koltai 2001), and the CDC working definition of “possible estuary-associated syndrome” (CDC 1999). Fishing areas were divided into grids on standardized maps that were given to the watermen, with map grid locations used to define where waterman had worked during the period covered by the log report. Participants received $50 compensation per quarter if they completed a minimum of five of six logs; the overall completion rate for the logs was 87.3%.

With the exception of 2001 (when post-season testing was delayed by IRB issues), participants received a neuropsychological screening battery preseason and postseason for 4 years. The neuropsychological screening battery was approximately 2 hr in length and was designed to assess a wide variety of cognitive functions that could potentially be altered by exposure. Measures of mood, effort, and other personality or psychiatric factors that could potentially interfere with cognitive performance were also included. Participants received $100 compensation for each testing session. The battery included the following components:

- Sensory and motor: Snellen test, Functional Acuity Contrast Test (Ginsburg 1993), smell test, and Lafayette Grooved Pegboard (Lafayette Instruments, Lafayette, IN)
- Attention, divided attention, and concentration: Wechsler Adult Intelligence Scale-III (WAIS-III) Digit Span (Wechsler 1997), Symbol Digit Modalities Test (Smith 1982), Trail-Making Test (Reitan 1992), Stroop Color-Word Test (Golden 1978), and WAIS-III Letter-Number Sequencing (Wechsler 1997)
- Memory: Rey Auditory Verbal Learning Test (Schmidt 1996), Rey-Osterrieth Complex Figure Test, Rey 15-Item Memory Test (Rey 1964), and recall (Meyers and Meyers 1995)
- Visual spatial and constructional: Rey-Osterreith Complex Figure, copy (Meyers and Meyers 1995), WAIS-III, Block Design (Wechsler 1997)
- Verbal fluency: Controlled Oral Word Association (Benton et al. 1994)
- Effort: Portland Digit Recognition Test (Binder 1993).

Measures of general intellectual functioning (Raven’s Standard Progressive Matrices; Raven et al. 1992) and reading proficiency (Wide Range Achievement Tests-3; Wilkinson 1993) were taken at the time of the first (baseline visit). Personality and mood were screened with the Profile of Mood States (McNair et al. 1971, 1992), Beck Depression Inventory II (Beck 1996), and the State Trait Anxiety Inventory (Spielberger 1983). The Brief Michigan Alcoholism Screening Test (Pokorny et al. 1972) and Alcohol Use Disorders Identification Test (AUDIT; Babor et al. 1989) were used to determine alcohol use and history, and a blood alcohol content screen was conducted via breathalyzer at the time of the exam. Finally, educational, occupational, neurological, psychiatric, and exposure histories were obtained via standardized interview.

### Environmental sampling

Water samples were collected for polymerase chain reaction (PCR)-based monitoring for the presence of *Pfiesteria* and other harmful algal bloom species during the period of this study (1999–2002) as part of an ongoing monitoring program by the Maryland DNR (Table 1). Samples collected by DNR staff during 1999 (*n* = 228), 2000 (*n* = 381), 2001 (*n* = 438), and 2002 (*n* = 387) were obtained from the lower eastern shore tributaries where the enrolled watermen worked.

The overlapping study participant work-area grids and Maryland DNR *Pfiesteria* sampling grids provided an opportunity to analyze study outcomes (reported symptoms and test results) with work in areas where *Pfiesteria* species were detected in the water column but did not provide certainty regarding the temporal overlap of work exposure and *Pfiesteria* detection. We therefore engaged willing watermen in a sampling protocol in the final two seasons of the study to further refine our correlation of exposure estimation with outcome measures. Under the waterman sampling protocol, potentially exposed cohort members from three general areas (Smith Island, mainland Somerset county, and Dorchester county) took water samples before departing their work area at the end of the day. In 2001, watermen collected samples on a biweekly basis (*n* = 426), and in 2002, on a weekly basis (*n* = 1,677).

Samples were collected in either 50-mL tubes (watermen) or 500-mL bottles (Maryland DNR) and fixed with 1% acidic Lugol’s solution (Vollenweider 1974). Once received in the laboratory, a 50-mL aliquot was centrifuged to pellet cells. Supernatant was removed, and total DNA was extracted using the Puregene Genomic DNA Isolation Kit (Gentra Systems, Minneapolis, MN). Real-time PCR assays specific for *Pfiesteria piscicida* and *Pfiesteria shumwayae* [recently renamed *Pseudopfiesteria shumwayae* (Litaker et al. 2005)] were performed as previously described (Bowers et al. 2000). We have previously demonstrated that Lugol’s-fixed specimens can be used to detect these organisms in environmental water samples without loss of assay sensitivity due to variations in sample processing and delivery times (Bowers et al. 2000).

### Statistical analysis

Over the course of the study, any given waterman participant could be exposed to *Pfiesteria* in one year and not exposed in another year. For this reason, we performed a preliminary statistical analysis for each study year separately before analyzing the pooled data. Statistical analyses for any given period of time involved comparisons among the following groups: watermen exposed to *Pfiesteria* (“exposed watermen”), watermen not exposed to *Pfiesteria* (“unexposed watermen”), and nonwaterman community residents (“community controls”). The watermen were classified as exposed or not exposed according to whether they spent time in waters that tested positive for *Pfiesteria* during the in-season period. Additional analyses were also conducted with watermen classified into four levels of exposure based on the amount of time spent working in waters that tested positive for *Pfiesteria*: no exposure, low exposure, moderate exposure, and high exposure.

For purposes of analysis, symptoms were grouped into five major categories (see Appendix): cognitive, gastrointestinal, irritation, pain, and respiratory. To determine whether *Pfiesteria* exposure was associated with symptoms of various kinds, between-category comparisons of symptom rates were made during each of the three periods described above. A longitudinal (three time points) Poisson regression model was fitted for each symptom category using the generalized estimating equation (GEE) method (Liang and Zeger 1986) with an offset corresponding to the time in each period covered by the subject’s symptom logs. The dependent variable was determined as the cumulative number of symptom episodes of a specific kind (skin, respiratory, etc.) reported by the subject in the period. The independent variables included indicator variables for the comparison groups, for the periods and for the period × group interactions. Relative risks (RRs) for *Pfiesteria* exposure and 95% confidence intervals (CIs) were determined from the estimated parameters of the model and their SEs. These analyses were conducted for each year separately and then for the 4 study years combined. Analyses were also conducted with *Pfiesteria* exposure as a binary (exposed/not exposed) variable and then with four levels of exposure.

To determine whether *Pfiesteria* exposure influenced neurocognitive test performance, between-group comparisons of test score means were made for the preseason and the postseason periods (as noted above, testing was not typically performed during the in-season period). A longitudinal (two time points) regression model was fitted for each neuro-cognitive test using the GEE method. The dependent variable was the *z*-score for a given test (standardization based upon initial test means and SDs for the nonwaterman controls). The independent variables included indicator variables for the comparison groups, for the periods, and for the period × group interactions, as well as the covariates age and education. Between-group standardized differences and 95% CIs were determined from the estimated parameters of the model and their SEs. As with the symptom data, the neuro-cognitive test data were analyzed with *Pfiesteria* exposure treated as a binary variable and then as levels of exposure.

## Results

### Environmental sampling

**Figure 2:**
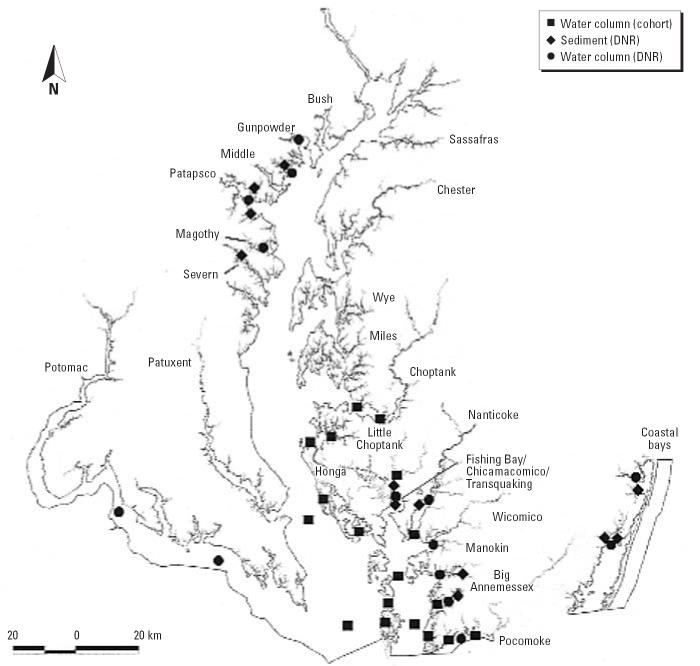
Locations within Chesapeake Bay and sample source (water column samples from cohort members vs. water column or sediment samples collected by DNR) for environmental samples positive for *P. piscicida* and *P. shumwayae*, 1999–2002.

We analyzed water samples collected in the study region by the Maryland DNR throughout the study period (Table 1). In 1999, 4.8% of 228 DNR-collected samples were positive for *P. piscicida*, whereas in 2000, 1.8% of 381 DNR-collected samples were positive. Rivers where *P. piscicida* was detected in the water column (Figure 2) included the Chicamacomico (10 of 11 samples in 1999 and 4 of 7 in 2000), the Pocomoke (1 sample in 1999, 2 samples in 2000), and the Big Annemessex (1 sample in 2000). *P. shumwayae* was not detected in either year. During 2001, *P. piscicida* was detected more frequently in DNR samples from the region (3.4%) than in waterman samples (0.9%) This degree of variation is partly influenced by more extensive DNR sampling of a particular watershed (the Chicamacomico and Transquaking rivers) where 10 of the 15 positive samples were collected during that year. Watermen tended not to work in this watershed, and few waterman-submitted samples were received from this area. Other rivers in which *P. piscicida* was detected in 2001 (DNR and waterman samples) included the Big Annemessex (*n* = 2), Manokin (*n* = 3), Choptank (*n* = 1), and Pocomoke (*n* = 1) rivers and Tangier Sound mainstem (*n* = 2). In 2001, *P. shumwayae* was not detected in waterman-submitted samples, but it was detected in 0.7% of DNR samples, all from the Pocomoke River. In 2002, *P. piscicida* was detected in 7% of samples collected by DNR and in 2.6% of waterman-submitted samples, a variation that we again attribute to heavier sampling in the Chicamacomico/ Transquaking region by DNR (19 of a total of 27 positive water samples were obtained in this watershed; six waterman samples collected in this system were positive). Other rivers in which *P. piscicida* was detected in 2002 (DNR and waterman samples) include the Tangier Sound region (*n* = 15), Chesapeake Bay main-stem (*n* = 4), and Honga (*n* = 3), Nanticoke (*n* = 3), Choptank (*n* = 2), Little Choptank (*n* = 2), Manokin (*n* = 4), Pocomoke (*n* = 11), and Wicomico (*n* = 1) rivers. In 2002, *P. shumwayae* was detected only in water specimens submitted by watermen (0.2%), and the organism was detected in the Pocomoke (*n* = 2), Honga (*n* = 1), and Little Choptank (*n* = 1) rivers and the mainstem of the bay (*n* = 1).

Through the course of the 4-year study, we observed a seasonal rhythm in the presence of detectable *Pfiesteria* zoospores in the water column (Figure 1). Many dinoflagellate species in the Chesapeake have characteristic bloom dynamics (e.g., *Prorocentrum minimum*; Tyler and Seliger 1978), and for *Pfiesteria* species, it appears that the organism is most prevalent in the water column during the late summer and early fall. In other investigations, we have demonstrated that *Pfiesteria* can be detected in sediments (presumptively as cysts) throughout the year (Bowers HB, Oldach DW, unpublished data).

### Symptom reporting

**Figure 3:**
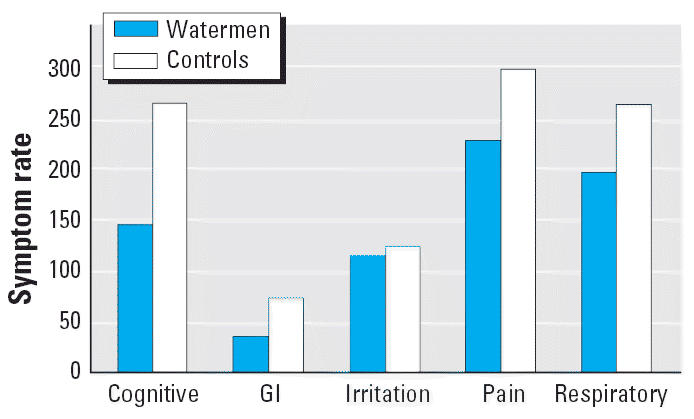
Symptom rates per 1,000 person-weeks, by major symptom categories, for watermen and controls, 1999–2002. GI, gastrointestinal.

Relative frequencies of reporting of each symptom category among watermen and community control persons are shown in Figure 3. Watermen as a group did not report symptoms with significantly greater frequency than did community controls, even after stratification by season (preseason/active season/postseason) and year. Similarly, there was no significant increase in the frequency of any symptom category, by season and year, when exposed watermen (those who had worked in areas where *Pfiesteria* had been detected) were compared with community controls.

When exposed watermen were compared with unexposed watermen (who had not worked in areas where *Pfiesteria* had been detected), there was greater variability in the results, with isolated increases in RR for specific symptom categories. For example, there was a significant increase in cognitive symptoms among exposed watermen during the active season (RR = 1.83; 95% CI, 1.22–2.70; *p* = 0.003) and postseason (RR = 4.63; 95% CI, 2.68–8.05; *p* < 0.001) in 2000; gastrointestinal symptoms, in contrast, showed an increase in the preseason (RR = 3.86; 95% CI, 1.67–8.70; *p* = 0.001) and active season (RR = 1.90; 95% CI, 1.10–3.18; *p* = 0.016) in 2000, but no increase in the postseason. Irritation symptoms showed an isolated increase in the preseason (RR = 2.45; 95% CI, 1.36–4.27; *p* = 0.002), and respiratory symptoms showed a slight increase through all three seasons: preseason (RR = 2.16; 95% CI, 1.40–3.26; *p* < 0.001), active season (RR = 1.82; 95% CI, 1.36–2.42; *p* < 0. 001), and postseason (RR = 1.96; 95% CI, 1.43–2.66; *p* < 0.001).

In no instance was there a consistent pattern of increases in multiple symptom categories in a single year, nor did we see a pattern of increase in any one symptom during the active season (when exposures to *Pfiesteria* would have been most likely to have occurred) across multiple years. In addition, no such patterns were seen regardless of whether watermen were compared with community controls, exposed watermen were compared with unexposed watermen, or four levels of exposure among watermen were compared. Although sample sizes were small, results were unaffected when the analysis was restricted to waterman-collected water samples or when data for *P. shumwayae* were included.

### Neuropsychological testing

Neuropsycho-logical data indicated no significant baseline differences between exposed and nonexposed watermen for age (*t* = 1.64, *p* = 0.11), years of education (*t* = 1.08, *p* = 0.28), or general intellectual functioning (Raven’s Standard Progressive Matrices total score; *t* = 0.72, *p* = 0.48). There were also no differences between the groups on measures of mood (Profile of Mood States total mood disturbance; *t* = 0.08, *p* = 0.94), anxiety (State Trait Anxiety Inventory; *t* = 0.76, *p* = 0.45), alcohol use (AUDIT; *t* = 0.39, *p* = 0.70), or malingering [Rey 15-Item Memory Test (Rey 1964); *t* = 0.50, *p* = 0.62].

When exposed and unexposed watermen were compared over time, we observed no significant differences in test performance between the groups on the Rey Auditory Verbal Learning Test or the Controlled Oral Word Association. In fact, 1999 was the only year in which significant differences were present between exposed and unexposed watermen on any of the key measures in the neuropsychological test battery. In 1999, there was a small but statistically significant increase in performance among exposed watermen in the Trail-Making Test, part B, in both pre-season (score difference = 0.62; 95% CI, 0.09–1.15) and postseason (score difference = 0.81; 95% CI, 0.26–1.37). Also in 1999, exposed watermen scored significantly higher on the Lafayette Grooved Pegboard dominant hand on preseason testing (score difference = 0.26; 95% CI, 0.01–0.51).

In no instance did exposed watermen show a pattern of neuropsychological decline in the postseason testing compared with controls. There were no alterations in psychological, psychiatric, or cognitive status. This finding was true for all tests, for all years, and in comparisons with both possible control groups (community controls and unexposed watermen).

## Discussion

Dinoflagellates in the genus *Pfiesteria* were first identified in the early 1990s by Burkholder et al. (1992) at North Carolina State University (Raleigh, NC) in association with fish kills in the Pamlico and Neuse estuaries. Two *Pfiesteria* species, *P. piscicida* and *P. shumwayae*, have been identified (Glasgow et al. 2001a, 2001b), although recent studies indicate that *P. shumwayae* should be placed in a separate genus, *Pseudopfiesteria* (Litaker et al. 2005). Numerous other “*Pfiesteria*-like organisms” have been characterized by both classical taxonomic and molecular methodologies (Steidinger et al. 2001).

Early studies from the Burkholder laboratory suggested that characteristic “punched out” skin lesions in fish were attributable to exposure to toxic forms of *P. piscicida* (Burkholder et al. 1992; Glasgow et al. 2001a, 2001b). However, the linkage of *Pfiesteria* species to lesioned fish has been highly controversial (Kane et al. 2000; Law 2001; Vogelbein et al. 2002). Current data indicate that most ulcerated lesions in menhaden (the fish most commonly affected) from the Chesapeake are due to a highly invasive fungal species, *Aphanomyces invadeans* (Blaser et al. 1999; Vogelbein et al. 2002). It has been hypothesized that fish-kill events attributed to *Pfiesteria*, and human health effects attributed to exposure to *Pfiesteria* blooms or laboratory cultures, are mediated by production of a toxic moiety by the organism. This has also been a matter of substantial scientific controversy, for full characterization of the presumptive hydrophilic toxin (tentatively named PfTx) has not been achieved (Berry et al. 2002; Burkholder et al. 2005; Fairey et al. 1999; Levin et al. 2003; Moeller et al. 2001; Vogelbein et al. 2002). This is an ongoing area of investigation for multiple laboratories, and confirmation of the existence of a *Pfiesteria* toxin falls outside the scope of the present epidemiologic investigation. Of note, an assay to detect putative *Pfiesteria* toxins in environmental samples is not available and was not available during the course of these studies.

Given uncertainty about the causal relationship between *Pfiesteria* species and fish lesions, the lack of specificity of both fish lesions and fish kills as a marker for the organism, and the absence of an assay for detection of the toxin in environmental samples, we elected to use molecular methods (PCR) developed in our laboratories (Bowers et al. 2000; Oldach et al. 2000; Rublee et al. 2001) to determine whether *Pfiesteria* was present in estuarine waters to which our cohort members were exposed. These assays have been validated by a number of investigators and have proven to be effective for monitoring *Pfiesteria* in the environment (Jakobsen et al. 2002; Rublee et al. 2001). We have previously demonstrated that the assay used for detection of *P. piscicida* in this study is equally effective for detection of *Pfiesteria* strains believed to have toxic and nontoxic phenotypes (in fact, these organisms have identical 18S ribosomal DNA sequences) (Tengs et al. 2003). Thus, the strategy we adopted for this 4-year field study to detect human health effects of recurring occupational exposure to an organism that may or may not actually make a toxin (that may or may not affect humans), and that may or may not cause fish kills and fish lesions, was to simply monitor for the organism itself, with an assay proven to be able to detect “toxic” strains. The assay we used was not quantitative, but we did not feel that adequate quantitative methods were available at the time we were doing the study. Although we cannot exclude the possibility that subtle differences were missed by reliance on qualitative data, use of quantitative data would have generated a number of additional uncertainties that would have made interpretation of this complex data set even more difficult.

Through the first 2 years of the study, screening was restricted to water samples collected by the Maryland DNR at designated sampling stations. When additional funding became available during the final 2 years of the study (2001 and 2002), we recruited watermen to collect specimens from their work sites as described above. For the first 2 years, the relatively large area encompassed by each of our map grid areas, coupled with a sampling frequency of once each 2 weeks, may not have optimally reflected the exposure of cohort members, despite the fact that this represented a substantial improvement over previously available methodologies (lesioned fish exposure and “*Pfiesteria*-like organism” counts on plankton microscopy). With the inclusion of waterman-collected samples from 2001 and 2002, our findings remained negative, with the added assurance that water sample data reflected authentic “exposure” (time-and place-matched sampling). It has been suggested that toxicity in *Pfiesteria* is restricted to a clonal subset of strains (Burkholder et al. 2001, 2005). Because our assay identified all organisms within the species, independent of possible toxicity, we cannot rule out the possibility that our failure to observe any human health effects resulted from the lack of “toxic” *Pfiesteria* in our study area during the 1999–2002 summers.

Despite these potential problems, this study represents the first systematic, multiyear effort to correlate human health effects with exposure to waterways where presence of *Pfiesteria* has been clearly documented. Given the large number of variables that were monitored, it is not surprising that we saw occasional differences between exposed and control populations; however, in no instance was there a consistent pattern of responses (either of reported symptoms or from formal neuro-psychological testing) that would suggest a health risk arising from occupational exposure to the estuarine environment. We saw no alterations in psychological or psychiatric status, in keeping with our observation that the initial group of persons exposed to the Pocomoke River were psychologically healthy with high energy, enthusiasm, and positive mood [i.e., there was no evidence that the initial symptom complex was related to functional or psychiatric factors (Tracy et al. 1998)]. Our exposure assessments, based as they were on molecular testing, were highly specific but may have lacked sensitivity. As noted above, the use of fish health (the occurrence of fish lesions or fish kills) is of uncertain validity as a marker for the organism but may highlight the presence of toxic strains in the environment, should such strains exist. In this context, the previously cited North Carolina studies (which used fish health as a marker for exposure) are reassuring in that they also failed to find a correlation between exposure and health (except for a possible correlation with reduced visual contrast sensitivity in the initial occupational prevalence study) (Moe et al. 2001; Swinker et al. 2001).

There is no question that persons exposed to the Pocomoke River in the summer of 1997 had profound, reversible (and well-documented) neuropsychological deficits (Grattan et al. 1998). Based on findings from laboratory-exposed persons (Schmechel and Koltai 2001), results of ongoing animal studies (Levin 2001; Levin et al. 2003), and studies that have begun to implicate specific neuro-receptors in the observed effect (El-Nabawi et al. 2000), it is plausible that *Pfiesteria*, in unique, isolated instances and/or in association with specific, unusually toxic strains, can cause human health effects. However, this study, in conjunction with similar studies from North Carolina and Virginia (Moe et al. 2001; Swinker et al. 2001), provides reassurance that, in the absence of an outbreak situation or the identification of a particularly toxic strain, the routine, occupational exposure to estuarine waters in which *Pfiesteria* is known to be present does not represent a significant human health risk.

## Figures and Tables

**Table 1:** Numbers of environmental samples collected, and number positive for *P. piscicida* and *P. shumwayae*, by year and source of sample (DNR vs. watermen).

	DNR samples			Waterman samples			
		No. positive (%)			No. positive (%)		
Year	No. of samples	*P. piscicida*	*P. shumwayae*	No. of samples	*P. piscicida*	*P. shumwayae*	Total samples Tested
1999	228	11 (4.8)	0	NA			228
2000	381	7 (1.8)	0	NA			381
2001	438	15 (3.4)	3 (0.7)	426	4 (0.9)	0	864
2002	387	27 (7)	0	1,677	43 (2.6)	5 (0.2)	2,064
Total	1,434	60 (4.2)	3 (0.2)	2,103	47 (2.2)	5 (0.2)	3,537

NA, not available; samples were not collected by watermen in the 1999 and 2000 seasons.

## Appendix: Symptoms for Which Biweekly Information Was Requested from Cohort Members (by Major category)

Cognitive

- Headache
- Confusion
- Problems concentrating
- Problems recalling tasks
- Problems recalling words
- Memory loss

Gastrointestinal

- Diarrhea
- Cramping belly pain
- Nausea or vomiting

Irritations

- Eye irritation
- Nasal irritation
- Skin irritation or burning
- Skin ulcers, lesions, or rash

Respiratory

- Cough
- Wheezing
- Shortness of breath
- Stuffy nose
- Sore throat

Pain

- Muscle cramps
- Joint pain
- Unusual fatigue or exhaustion
